# New Insights on Genetic and Morphological Divergence Among a *Buthus* Species Complex From Tunisia With the Identification of a New Species

**DOI:** 10.1002/ece3.72556

**Published:** 2025-11-30

**Authors:** Sarra Hajri, Lilia Bahri, David James Harris

**Affiliations:** ^1^ Research Laboratory “Biodiversity, Parasitology and Ecology of Aquatic Ecosystems” (LR18ES05), Faculté Des Sciences de Tunis University of Tunis El Manar Tunis Tunisia; ^2^ BIOPOLIS Program in Genomics, Biodiversity and Land Planning, CIBIO‐InBIO, Campus Agrário de Vairão Universidade do Porto Vairão Portugal

**Keywords:** *B. saidnouirai* sp. *nov*, *Buthus*, COI barcoding, morphology, taxonomy, Tunisia

## Abstract

The taxonomy of the scorpion genus *Buthus* is complex due to the considerable increase in newly reported species, their high degree of similarity, and consequently, the great difficulty in their morphological differentiation. Tunisian species are not exempt from this issue, with several references highlighting the need for taxonomic revisions. This study integrates DNA sequence data and morphological assessments to investigate the diversity present in Tunisia and to provide morphological details that facilitate species identification. The results show that most Tunisian specimens are distributed within two clades. One clade comprises four subclades corresponding to *B. tunetanus* Herbst, 1800, *B. paris* C. L. Koch, 1839, *B. chambiensis* Kovařík 2006 and a southern group corresponding probably to *B. lourencoi* Rossi, Tropea & Yağmur, 2013. The second clade represents a new species described in this study as *B. saidnouirai* Hajri, Bahri & Harris, *sp. nov*. No evidence of *B. dunlopi* Kovařík 2006 have been recorded in the studied samples. Distances between all five species exceed the minimum divergence thresholds for *Buthus* species. The greatest distance was observed between *B. saidnouirai. sp. nov*. and the southern group, while the smallest distance was between *B. tunetanus* and *B. paris*. Although the genetic differences revealed considerable divergence of the new group from the four remaining species, the morphological assessment did not identify the same pattern. These five species demonstrate a morphological shape gradient in which *B. paris* and the southern group represent the two extremes, with *B. paris* being the most ornamented and the latter the least. The new species presents an intermediate morphology. The geographic distributions of the five reported species are discussed in this work according to the topography and orography of the region. Additional lineages known from Algeria may also enter the western fringes of Tunisia.

## Graphical abstract

This work presents a revision of Tunisian species within the genus *Buthus*. By combining molecular and morphological analyses, we describe a new species and we better define the distribution area of all the recorded species and provide new morphological criteria that facilitate their identification and differentiation.

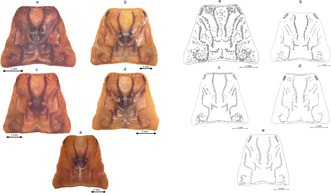

## 1 Introduction

*Buthus* is a genus of scorpions belonging to the Buthidae family, proposed in Leach (1815) with a single diagnostic criterion. His definition was considered by many taxonomists as overly simplistic, as it led to a rapid increase in the number of species belonging to this genus (100 species up to 1950) that are very different from the type species description (Vachon 1952; Levy and Amitai 1980; Lourenço 2002). Following the major revision made by Vachon (1952) (Leach 1815), the taxonomy of this genus was reviewed, and only four species were assigned to it. By 2017, the number of accepted species had increased to 52 (Sousa et al. 2017), and since then the number of reported species has continued to increase, reaching until now 79 species in the world.

Within the Tunisian territory, four species of *Buthus* have been reported based on morphological criteria: *B. tunetanus* Herbst, 1800, *B. paris* C. L. Koch, 1839, *B. dunlopi* Kovařík 2006, and *B. chambiensis* Kovařík 2006. The first two species ( *B. tunetanus* and *B. paris*) were considered by Vachon (1952) as two subspecies of *B. occitanus* and were elevated to specific rank by Lourenço (2003). *B. tunetanus* has the widest distribution, inhabiting the majority of plateaus and plains of Tunisian territory, while *B. paris* inhabits the forest and mountain environments of northern Tunisia without a precise delimitation of their distribution area. The other two species are endemic, respectively to the Remeda and Chambi regions and were only recognized in 2006 (Kovařík 2006). However, in recent years, across the majority of studies of *Buthus* from Tunisia, authors report problems with assigning individuals to a specific species and have often only referred to the genus rank by identifying them as *Buthus* sp. (Sousa et al. 2010; Pedroso et al. 2013; Klesser et al. 2020). The lack of detailed delimitations of the ranges of described species, compounded by the absence of the type series of *B. tunetanus* and *B. paris* which have been reported as lost since the last century (details in Sousa et al. 2017), does not help make species identification less doubtful. These issues reveal the great difficulty of morphological discrimination of *Buthus* species. In similar cases, when the studied taxa are cryptic species or present high phenotypic plasticity, taxonomists often employ molecular approaches to enable taxon recognition alongside a morphological assessment (Hebert et al. 2003).

Globally, molecular systematic research regarding the *Buthus* genus has been carried out employing a variety of approaches. Various studies, particularly in the Iberian Peninsula and northeast Africa, using predominantly mitochondrial DNA sequences, identified high levels of genetic diversity and indicated this area was a key region for diversification (Sousa et al. 2010, 2012; Pedroso et al. 2013; Klesser et al. 2020; Gantenbein and Largiadèr 2003; Habel et al. 2012; Husemann et al. 2012). While other markers, such as the slower evolving nuclear 18S rRNA (Gantenbein and Largiadèr 2003) and mitochondrial 12S and 16S rRNA (Pedroso et al. 2013) were also employed, the most widely used marker was partial CO1 gene sequences.

This mitochondrial marker (COI) is characterized by its high mutation rate which makes it useful in identifying specimens to the species level in many taxa (Goodman et al. 2021). Indeed, DNA barcoding consists of the analysis of diversity among sequences of a standardized region, and typically targets the COI gene to assist in species recognition and discrimination (Hebert et al. 2003; Wilson 2010). Its effectiveness in taxonomic purposes has been demonstrated in many scorpion species such as *Androctonus crassicauda* and *Scorpio maurus* (Toprak et al. 2019), *Hemiscorpius lepturus* (Jolodar 2019), *Leiurus quinquestriatus*, *Androctonus amoreuxi*, *Orthochirus innesi*, and *Buthacus leptochelys* (Mohammed‐Geba et al. 2021), and as part of an integrated taxonomic approach has been used to revise the European species of *Buthus* (Blasco‐Aróstegui et al. 2025).

In a phylogenetic context, some authors have attempted to sequence this gene in some Tunisian *Buthus* specimens as part of assessments of larger geographic regions such as the Maghreb or the Mediterranean basin. Prior to this study, mitochondrial COI DNA from 27 *Buthus* specimens from Tunisia was sequenced (Sousa et al. 2010, 2012; Klesser et al. 2020; Gantenbein and Keightley 2004). They were all identified as *Buthus* sp., except for four individuals identified as *Buthus tunetanus*, reflecting the lack of systematic knowledge on these species in Tunisia and appealing for further taxonomic investigations to be done. Hence, this study aimed to reexamine the taxonomy of Tunisian *Buthus* scorpions based on a combination of COI sequences and morphological assessments and using a large sample of specimens distributed across the Tunisian territory, encompassing both recently described species' type localities and unexplored areas. Our main goal was to provide new insights into the identification of *Buthus* species occurring in Tunisia, which may help understand the diversity and evolution of this genus.

## 2 Materials and Methods

### 2.1 Sampling

**FIGURE 1:**
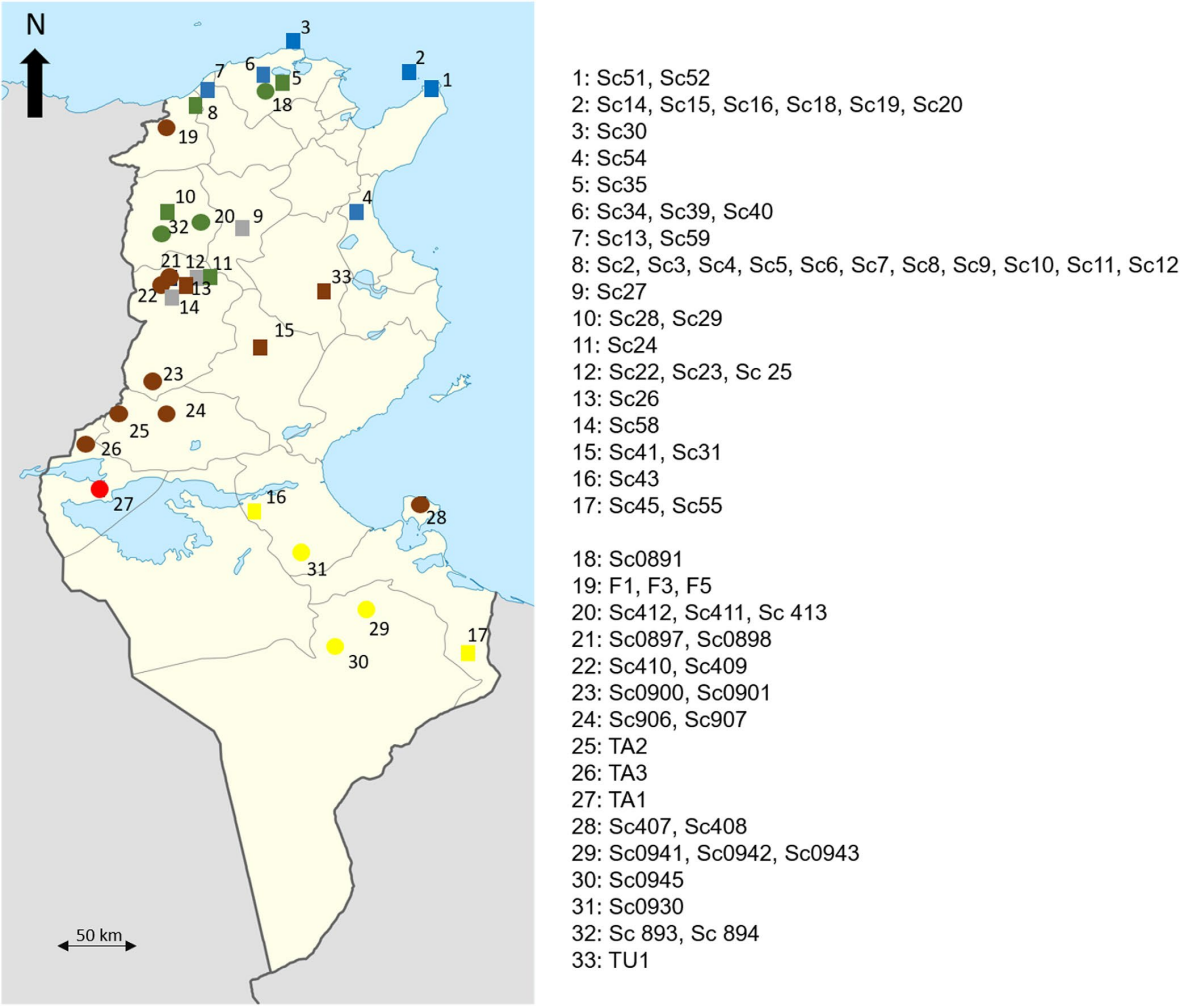
Geographical distribution of Tunisian samples. Squares represent specimens for which new sequences were generated in this study, while circles denote previously published specimens. The colors indicate different lineages: G1 (Green), G3 (Gray), D (Blue), G4 (Brown), G2 (Yellow), and Red for the Tunisian sample of Clade C as shown in Figure 2.

In total, our study included 81 *Buthus* specimens; 43 of these specimens were specifically sampled in this work. Scorpions were captured during night prospection, by using UV light and have been stored immediately in 96% ethanol (42 specimens from 17 Tunisian localities and one specimen from Egypt). The remaining 38 specimens (27 from 16 Tunisian localities and 11 from different Algerian localities) are stored mainly in the CIBIO Scorpion Collection, and their COI sequences were downloaded from GenBank. In total, this study included 69 Tunisian specimens from 33 different localities (Figure 1). As an outgroup for the phylogenetic analysis, one sequence of *Androctonus mauritanicus* published on GenBank has been included. Further information is provided in Table S1.

### 2.2 Molecular Analysis

DNA was isolated from the muscle of one leg using the high‐salt precipitation method (Sambrook et al. 1989). Employing the primers LCO‐1490 and HCO‐2198 (Folmer et al. 1994), a fragment of COI was amplified using a conventional polymerase chain reaction (PCR) approach. This was performed in an 11 μL volume reaction consisting of 7 μL of MyTaq mix, 2 μL of water, 0.5 μL of each primer (10 μM concentration) and 1 μL of DNA. The PCR program was: initial denaturation step at 95°C for 10 min, followed by 40 amplification cycles of 30 s denaturation at 93°C, 1 min annealing at 55°C and 45 s elongation at 72°C. Cycling was terminated by a final elongation step at 72°C for 10 min. The PCR's success was assessed using gel electrophoresis. Positive samples were purified using a combination of shrimp alkaline phosphatase and exonuclease I (ExoSap). This was followed by a sequencing reaction performed in a 10 μL volume reaction consisting of 7 μL of water, 1 μL of TRR buffer, 0.5 μL of LCO‐1490 primer, 0.5 μL of TRR and 1 μL of DNA from the PCR cleaned product. After running sequencing reactions on a thermocycler, Sephadex spin column cleaning was conducted followed by drying the reaction in a thermocycler for 25 min. 15 μL of Formamide was then added to each sample before running them through an ABI capillary electrophoresis system.

### 2.3 Molecular Data Analysis

Sequences for the corresponding region of COI from *Buthus* species were downloaded from GenBank. The alignment of these sequences with the newly generated sequences was conducted using MEGA 11 (Tamura et al. 2021). Due to different lengths of sequences for some sequences from GenBank, two datasets were assessed: the first with 82 sequences of 351 bp, and another with a subset of 51 sequences with 579 bp. The most appropriate evolutionary model was identified using the online resource PhyML‐ATGC (Montpellier Bioinformatics), and then a maximum likelihood tree was generated with support estimated using 1000 bootstraps (Guindon et al. 2005). MrBayes 3.2.7 was employed to estimate a Bayesian tree, using the same model of evolution, and run for 10 million generations, with the first 25% of trees excluded as burn‐in and visualized using Fig Tree v1.4.4 (Ronquist et al. 2012; Rambaut 2018).

Kimura 2‐parameter (K2P) distances between the different identified phylogenetic groups were calculated using MEGA 11 (Tamura et al. 2021). Reported distances were then compared with those between known *Buthus* species for this region of COI, by assessing 120 sequences from GenBank that were assigned to 15 different species of *Buthus*.

For molecular species delimitation we used the ASAP approach (Assemble Species by Automatic Partitioning; Puillandre et al. 2020). This method uses pairwise genetic distances to rank potential species partitions from a single locus sequence alignment. The barcode gap widths and the groups' likelihood of being panmictic species are used to obtain a composite score and rank (Puillandre et al. 2020).

### 2.4 Morphological Analysis

The morphological analysis was performed using the characters used in scorpion taxonomic classification (Vachon 1952; Lourenço et al. 2018; Teruel and Turiel 2020; Vahidinia et al. 2025). Under a stereomicroscope with an ocular micrometer all these characters were checked for 60 Tunisian specimens: the 42 newly collected ones and 18 from the collection at CIBIO. A comparative morphological description between Tunisian species was then developed. High‐quality photographs of the discriminating characters, were taken with a camera stereomicroscope and examined using Pixlr Editor. To simplify and highlight the intended characters, they have also been drawn using a drawing tube plugged into the stereomicroscope.

## 3 Results

**FIGURE 2:**
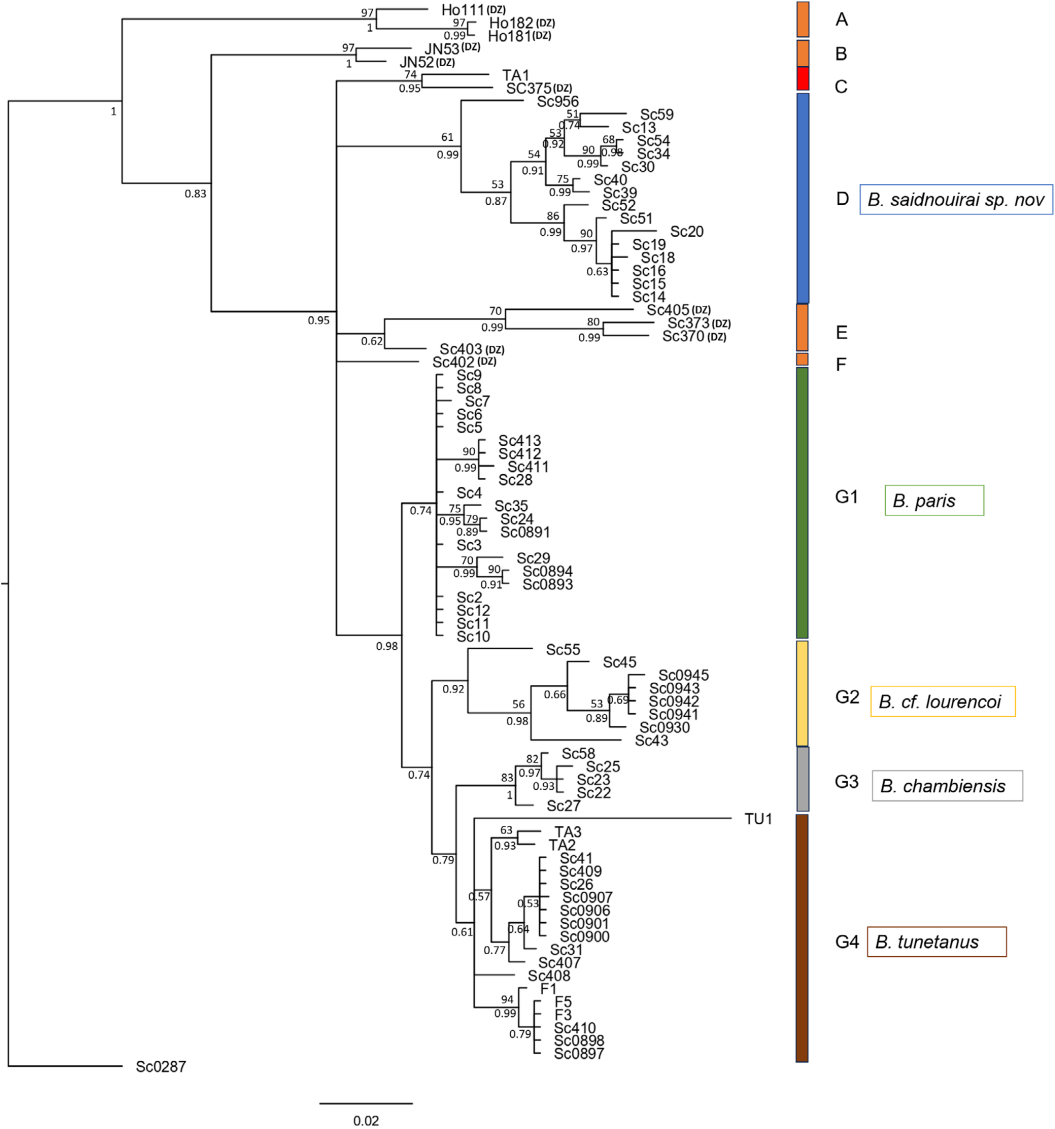
A phylogenetic tree showing the relationships among *Buthus* specimens as determined through a Bayesian approach. The numbers at the nodes below the branches indicate Bayesian posterior probabilities (BPP) derived from Bayesian inferences, while the numbers at the nodes above the branches represent bootstrap percentages obtained from maximum likelihood (ML) analysis (only bootstrap values over 50% are indicated).

Forty‐three new CO1 sequences from this study were combined with 38 sequences of *Buthus* species from GenBank and one outgroup, forming a data set of 82 sequences of 351 bp. All new sequences were submitted to GenBank (Accession numbers to be included upon acceptance). The most appropriate model was identified as the HKY85 + G + I, and the result of the Bayesian analysis with this model is shown in Figure 2. In this estimate of relationships, specimens from Algeria are scattered over different clades (A, B, C, E and F), revealing a wide genetic diversity that requires a greater sampling effort to be resolved. For Tunisian specimens, only TA1 (sampled from central western Tunisia; Figure 1) forms a robust clade, C (Bayesian posterior probability BPP = 0.9541) with an Algerian specimen (Sc0375), which was sampled near the Tunisian frontier (central Tunisian side). All remaining Tunisian specimens are spread among two clades. One of these clades comprises four subclades (G1, G2, G3, and G4). According to their geographic locations, G1, G3, and G4 would correspond to *B. paris*, *B. chambiensis* and *B. tunetanus* , respectively. G2 covers the south of Tunisia which contains the type locality of *B. dunlopi* but with a much wider distribution range. The other clade, D, which is identified here for the first time, is made up of all the other Tunisian specimens that are from coastal and humid regions. This new clade D also contains an individual from Egypt (Sc 956) which is also from a coastal location, Matrouh. Groups D, G1, G2, and G3 are supported as monophyletic groups, with Bayesian posterior probabilities exceeding 0.8 (Figure 2). The same pattern was observed when considering only 51 of the complete set of 82 sequences based on a longer fragment of 579 bp.

Distances between identified *Buthus* species in GenBank range from 2.51% to 14.61%, for the 351 bp targeted region. The genetic distances between our five groups for this same DNA region are reported in Table 1. All these distances are greater than the minimum divergence threshold (2.51%). G3 and G2 exhibit the highest divergence (9.76%) with the new group (D) while the lowest divergence is between G4 and G1 (3.41%). This same pattern of distances is also observed among the set of sequences with longer fragments (579 bp) (Table 1). The new group shows the highest distances compared with the groups G1–G4, which matches the distribution area of the four defined species in Tunisia.

**TABLE 1:** Mean distances between Tunisian clades (Above diagonal: 579 bp; below diagonal: 351 bp) (intraclade distances are between brackets after the clade name) (minimum–maximum distance values are between brackets after mean distances).

	G1 (0.01)	G3 (0.00)	D (0.03)	G4 (0.01)	G2 (0.03)
G1 (0.01)	—	0.0390 (0.030–0.0511)	0.0776 (0.061–0.098)	0.0325 (0.023–0.039)	0.0394 (0.024–0.054)
G3 (0.01)	0.0438 (0.035–0.056)	—	0.0926 (0.058–0.093)	0.0280 (0.013–0.03)	0.0405 (0.023–0.042)
D (0.03)	0.0792 (0.056–0.111)	0.0943 (0.076–0.121)	—	0.0844 (0.051–0.089)	0.0807 (0.050–0.861)
G4 (0.02)	0.0334 (0.014–0.094)	0.0404 (0.030–0.090)	0.0859 (0.045–0.101)	—	0.0366 (0.026–0.041)
G2 (0.02)	0.0503 (0.017–0.065)	0.0594 (0.031–0.056)	0.0834 (0.066–0.112)	0.0508 (0.027–0.072)	—

The ASAP analysis, with an ASAP‐score of 10.00 and *p*‐value of 9.521e‐01 (partitions on the right in the Appendix S1), shows two robust subsets: one grouping together the four groups approximately matching the distribution area of the four known species, and the other corresponding to a new group. The nine other partitions provided by ASAP (Appendix S1) yielded suggestions with a large number of subsets, the smallest containing 10 species in Tunisia. However, none of the proposals grouped the new cluster with the rest of the *Buthus* groups. This further indicates that the new form is divergent from the four other groups, which reinforces the recognition of a new species of *Buthus* in Tunisia.

**FIGURE 3:**
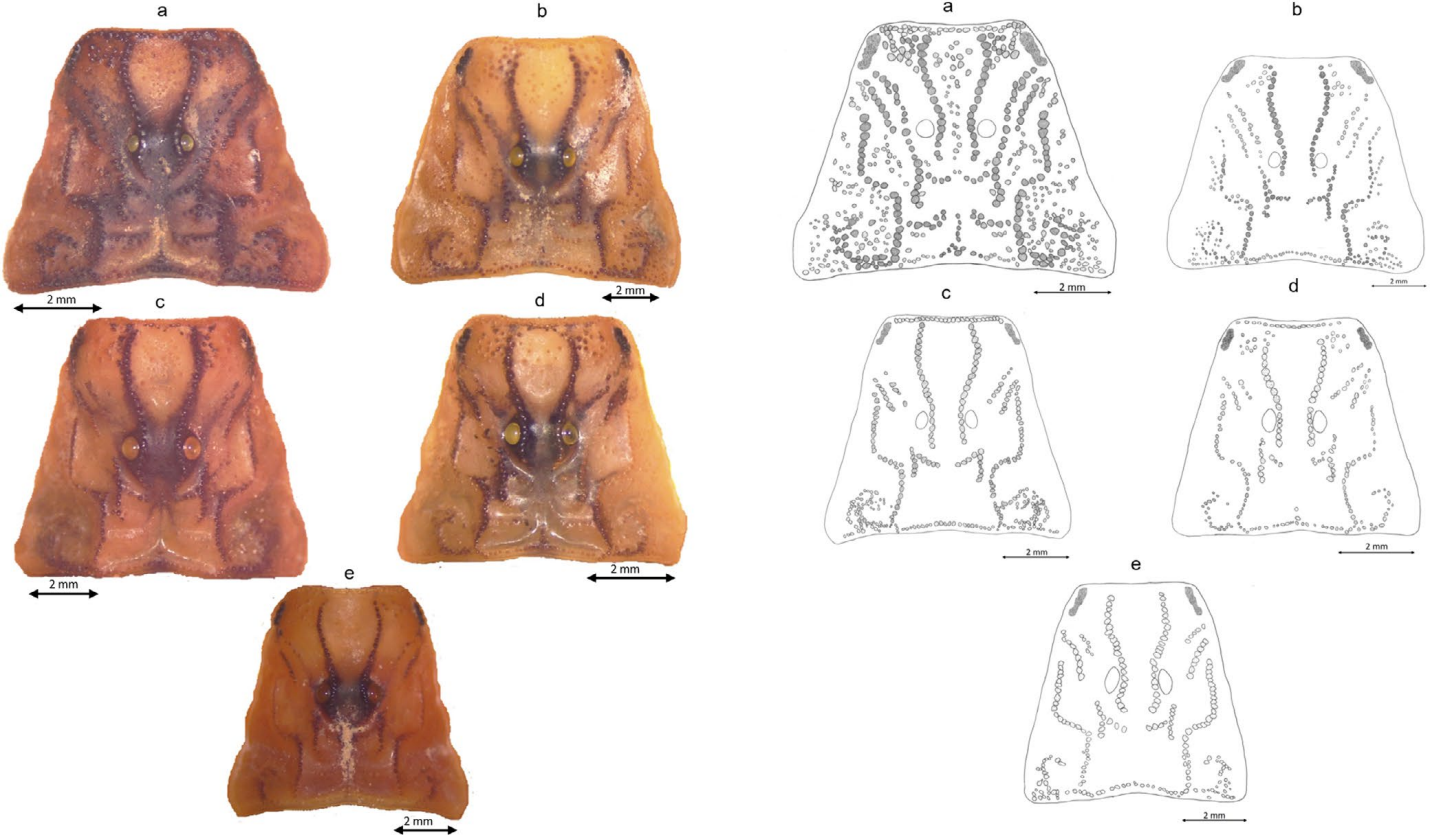
Carapace drawing for the five Tunisian species: (a) Carapace drawing of G1; (b) Carapace drawing of G3; (c) Carapace drawing of D; (d) Carapace drawing of G4; (e) Carapace drawing of G2.

**FIGURE 4:**
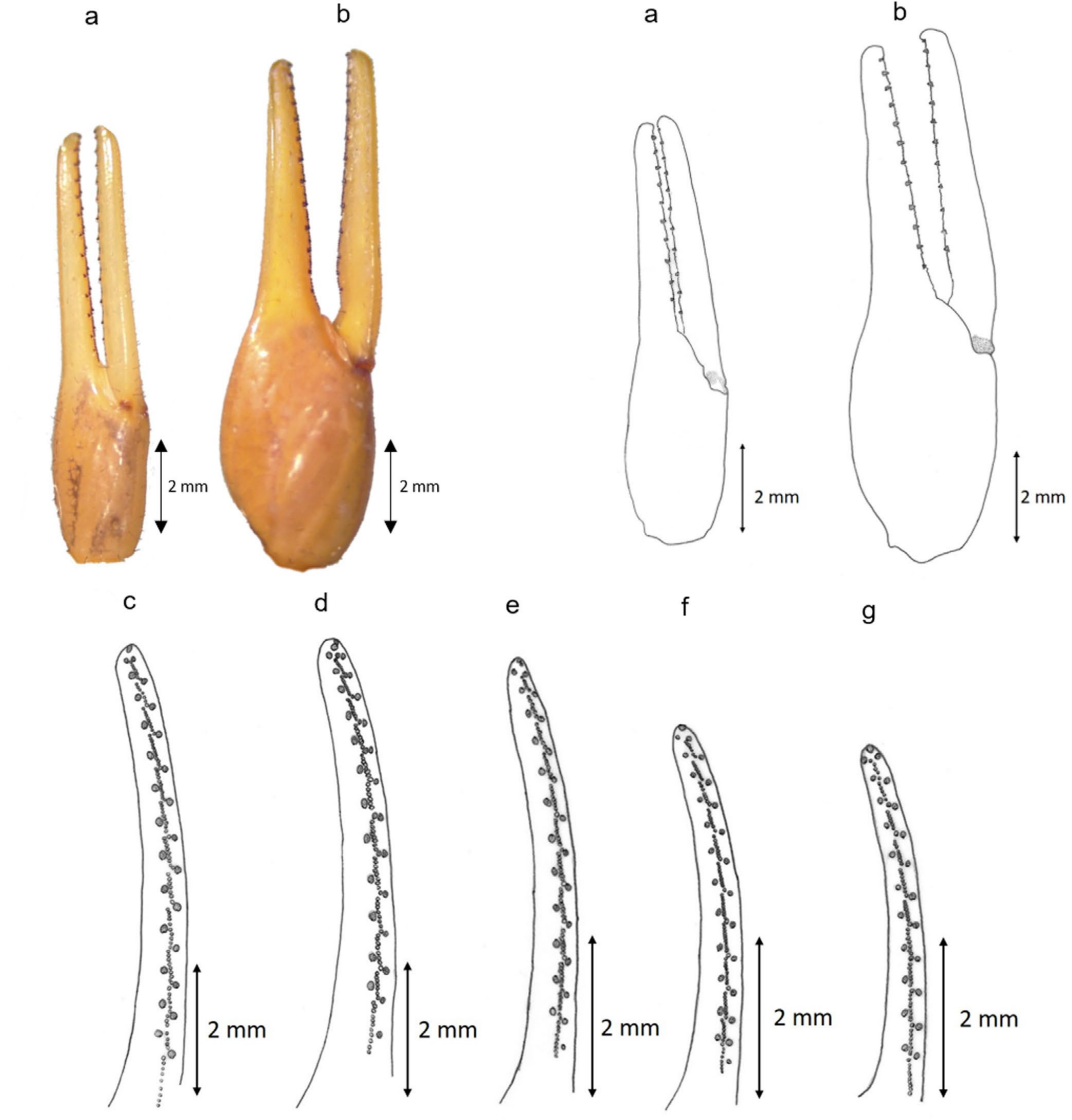
Manus and movable finger's rows of granules drawing for the five Tunisian species: (a) Manus drawing for G4 and G2; (b) Manus drawing for G1, G3 and D; (c) movable finger's rows of granules drawing for G1; (d) movable finger's rows of granules drawing for G3; (e) movable finger's rows of granules drawing for D; (f) movable finger's rows of granules drawing for G4; (g) movable finger's rows of granules drawing for G2.

**FIGURE 5:**
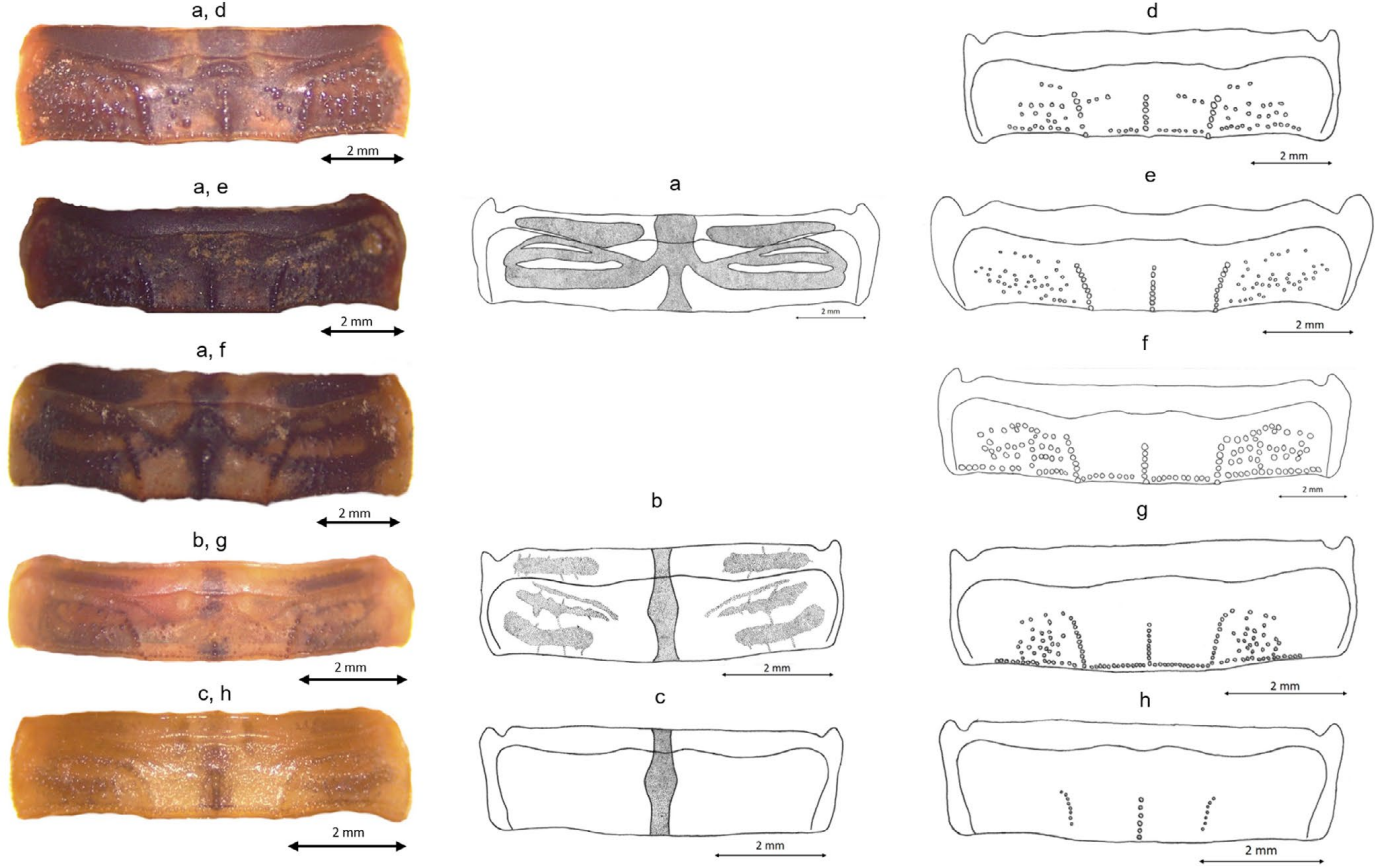
Tergites drawing for the five Tunisian species: (a) Bands drawing for G1, G3 and D; (b) Bands drawing for G4; (c) Bands drawing for (G2); (d) Granulation for G1; (e) Granulation for G3; (f) Granulation for D; (g) Granulation for G4; (h) Granulation for G2.

**FIGURE 6:**
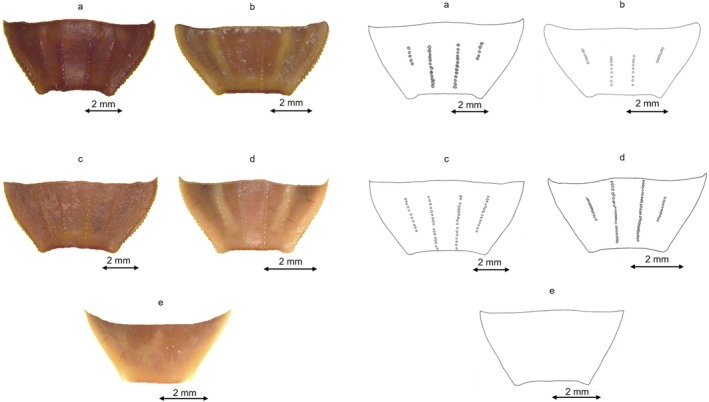
Sternite VII drawing for the five Tunisian species: (a) Sternite VII drawing for G1; (b) Sternite VII drawing for G3; (c) Sternite VII drawing for D; (d) Sternite VII drawing for G4; (e) Sternite VII drawing for G2.

**FIGURE 7:**
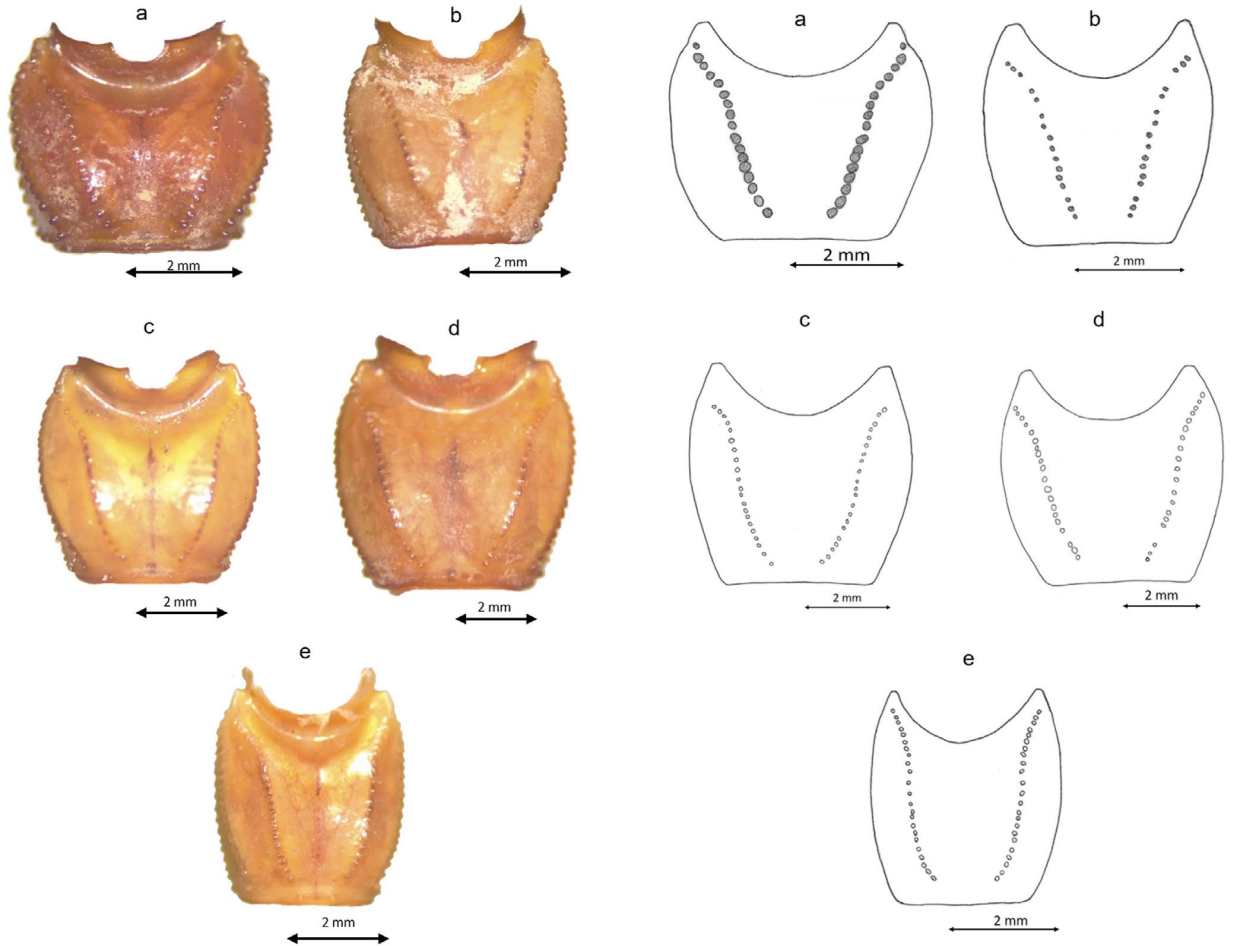
First metasomal segment drawing for the five Tunisian species: (a) Dorsal first metasomal segment for G1; (b) Dorsal first metasomal segment for G3; (c) Dorsal first metasomal segment for D; (d) Dorsal first metasomal segment for G4; (e) Dorsal first metasomal segment for G2.

**FIGURE 8:**
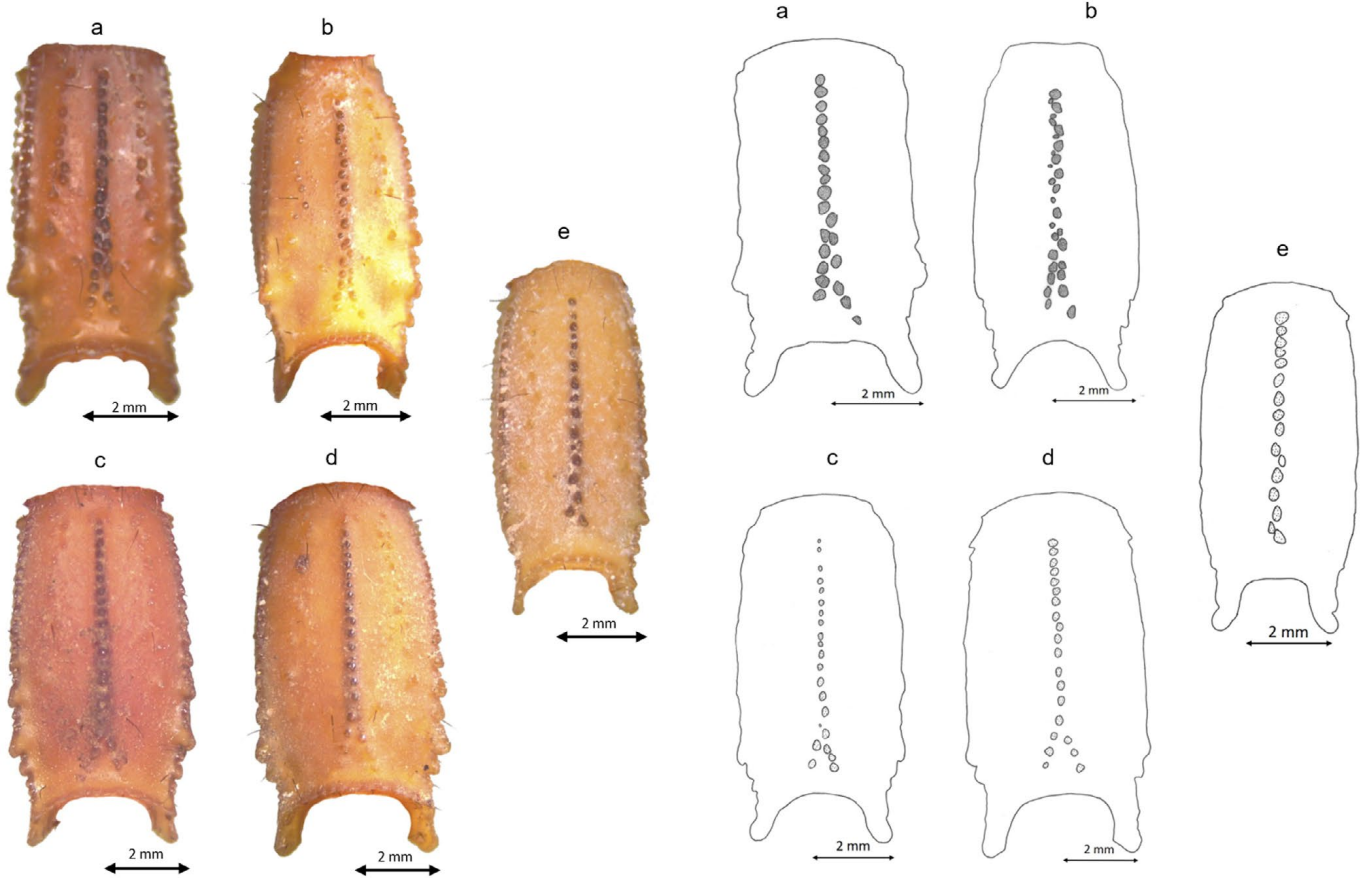
Fifth metasomal segment drawing for the five Tunisian species: (a) Ventral fifth metasomal segment for G1; (b) Ventral fifth metasomal segment for G3; (c) Ventral fifth metasomal segment for D; (d) Ventral fifth metasomal segment for G4; (e) Ventral fifth metasomal segment for G2.

**FIGURE 9:**
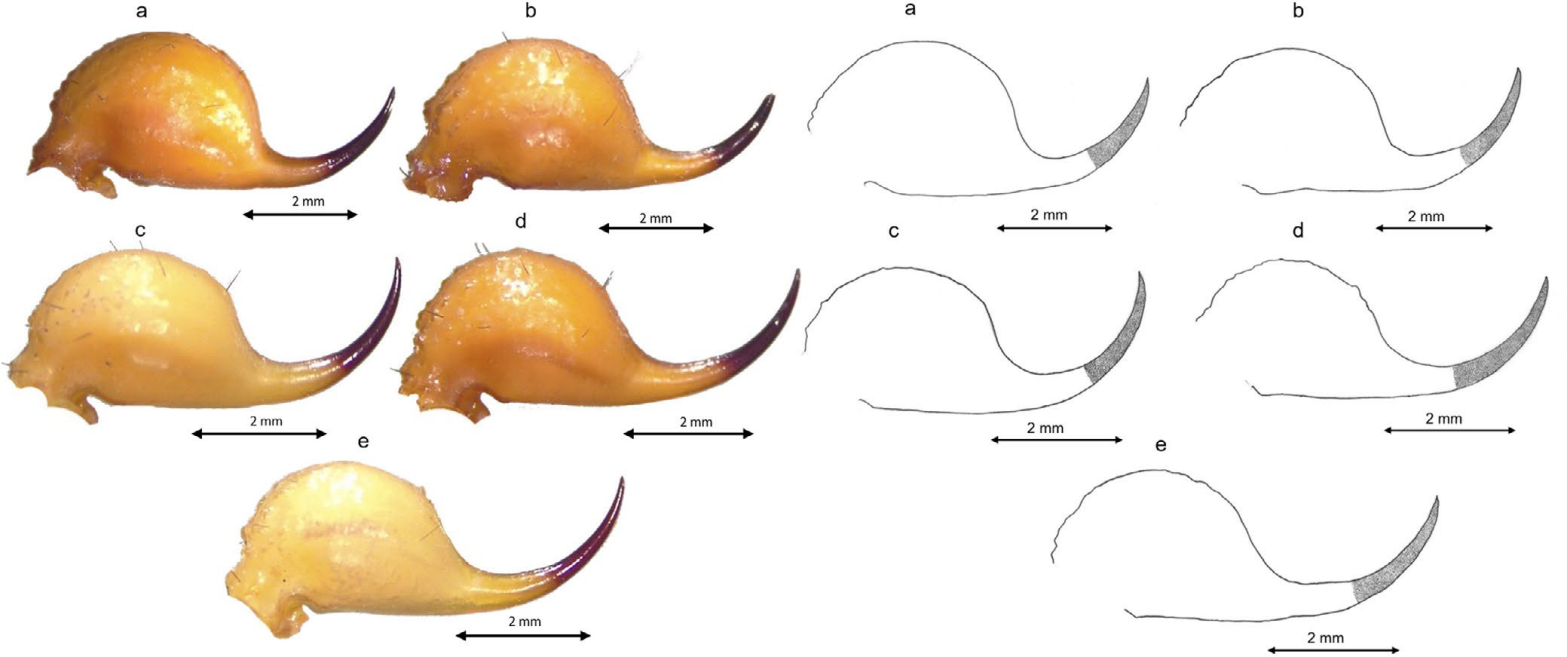
Telson drawing for the five Tunisian species: (a) Telson drawing for G1; (b) Telson drawing for G3; (c) Telson drawing for D; (d) Telson drawing for G4; (e) Telson drawing for G2.

Although the genetic differences revealed a considerable divergence of the new group from the four remaining groups, the morphological assessment did not identify a similar pattern. Rather, these five groups, presumably corresponding to five species, demonstrate a sort of morphological shape gradient as seen in the carapace (Table 2 and Figure 3), chela (Table 2 and Figure 4), mesosoma (Table 3, Figures 5 and 6), metasoma (Table 4 and Figure 7), and telson (Table 3, Figures 8 and 9). G1 and G2 present the two extremes of this gradient. Overall, G1 is the most ornamented, having the most prominent granules, the widest forms (carapace and metasoma), the highest number of rows of granules on the movable finger and the shortest aculeus, while G2 presents as the least ornamented, having the least prominent granules, the longest forms (carapace and metasoma), the lowest number of rows of granules on the movable finger and the longest aculeus. The new *Buthus* species presents an intermediate morphology. Clade G4, lies between G2 and the new species, while clade G3 lies between G1 and the proposed new species.

**TABLE 2:** Morphological differences of carapace and chela between the 5 species of *Buthus* in Tunisia.

	Character	G1	G3	D	G4	G2
Carapace	Shape	Stocky (noticeably wider than long) Mean (Length/width) = 0.79 ± 0.01 (min = 0.76; max = 0.81)	Stocky (noticeably wider than long) Mean (Length/width) = 0.77 ± 0.01 (min = 0.76; max = 0.79)	Tends to be elongated (slightly wider than long) Mean (Length/width) = 0.90 ± 0.03 (min = 0.85; max = 0.95)	Tends to be elongated (slightly wider than long) Mean (Length/width) = 0.96 ± 0.04 (Min = 0.89; max = 0.98)	Elongated Mean (Length/width) = 1.02 ± 0.01 (Min = 0.98; max = 1.03)
	Carinae's granules	Highly protruding	Highly protruding	Well protruding	Protruding	Lowly protruding
	Line (carinae in anterior part between the two lyres)	Present	Absent	Absent	Absent	Absent
	Placement of the lateral eyes in regard to the highest curvature line between the two carinae dividing the two median eyes	Highest curvature line cuts the lateral eyes	Highest curvature line under the lateral eyes	Highest curvature line under the lateral eyes	Highest curvature line under the lateral eyes	Highest curvature line under the lateral eyes
	Eyes shape	Circular	Circular	Oval	Oval	The most oval
Chela	Shape	Large and Robust Mean (Length/width) = 3.7 ± 0.2 (min = 3.3; max = 4.0)	Large and Robust Mean (Length/width) = 3.5 ± 0.2 (min = 3.3; max = 3.8)	Large and Robust Mean (Length/width) = 3.7 ± 0.1 (min = 3.5; max = 4.0)	Thin Mean (Length/width) = 4.3 ± 0.1 (min = 4.1; max = 4.5)	Thin Mean (Length/width) = 4.3 ± 0.2 (min = 4.2; max = 4.5)
	Rows of granules of movable finger	13	12–13	12	11–12	11

**TABLE 3:** Morphological differences of the mesosoma between the 5 species of *Buthus* in Tunisia.

	Character	G1	G3	D	G4	G2
Tergites	Basic color	Light or dark	Light or dark	Light	Light	Light
	Bands	An axial band with sagittal bands well traced and delimitated (clearly observable only on specimens with light basic color)	An axial band with sagittal bands well traced and delimitated (clearly observable only on specimens with light basic color)	An axial band with sagittal bands well traced and delimitated	An axial band with sagittal bands that are little mottled and not well delimitated and traced	Presence only of an axial band
	Granules in the lateral and axial carinae	Granules are big and all have the same size	Granules are mid‐sized and all have the same size	Granules are big and all have the same size	Granules are mid‐sized or small but all have the same size in the same specimen	Granules in the lateral carinae are small, while those in the axial carinae are slightly bigger
	Granules next to lateral carinae	Smaller than lateral carina granules	Smaller than lateral carina granules	Same size as carina granules	Same size as carina granules	Practically invisible
Sternites VII	Carinae	Two well‐marked pairs	2 pairs	2 pairs	2 pairs	Invisible
	Carinae granules	Granules are big, black and strongly protruding	Granules are big or mid‐sized	Granules are small or mid‐sized	Granules are small or mid‐sized having the same color of the sternite	—
	Anterior limit of both carinae	Same level	Exterior carinae higher than interior carinae	Same level	Interior carinae higher than exterior carinae	—
	Length of interior carinae	¾ of the sternite length	Less than ¾ of sternite length	A little more than ¾ of the sternite length	Along the sternite	—

**TABLE 4:** Morphological differences of the metasoma and telson between the 5 species of *Buthus* in Tunisia.

	Character	G1	G3	D	G4	G2
Metasoma	Carinae granules	Granules are big and strongly protruding	Granules are small and strongly protruding	Granules are small or mid‐sized and lowly protruding	Granules are small and lowly protruding	Granules are small and lowly protruding
	Size of the 1st segment	Wider than it is long	Length and width are almost equal	Length and width are almost equal	Length and width are almost equal	Longer than it is wide
	Size of the granules in the ventral face of the 5th segment's median carinae	Same size along the carinae	Along the carina there is a small granule next to the big granule	Granules increase in size from top to bottom	Same size along the carinae	Same size along the carinae
	Posterior extremity	Bifurcated	Bifurcated	Bifurcated	Bifurcated	No bifurcated
Telson	Vesicle	Bulbous	Bulbous tending to be Large	Bulbous tending to be Large	Bulbous tending to be Large	Large
	Vesicle versus aculeus	Aculeus shorter than the vesicle	Aculeus shorter than the vesicle or of equal length	Having almost the same length	Aculeus longer than the vesicle or of equal length	Aculeus longer than vesicle
	Aculeus curvature	High	High	High	Low	Low
	Position of the aculeus extremities in regard to the vertical tangent that passes through the vesicle's highest point	Aculeus extremity under the tangent	Aculeus extremity under the tangent	Aculeus extremity hits the tangent	Aculeus extremity under the tangent	Aculeus extremity under the tangent

Based on the integrated morphological and mtDNA sequence data, we here propose this form as a new species, *B. saidnouirai* Hajri, Bahri & Harris, sp. nov. (Zoobank LSID: urn:lsid:zoobank.org:act:49F11785‐55C1‐4DAD‐B95B‐CACCB6FA9499).

### 3.1 Taxonomy

Family: Buthidae E. Simon, 1879 (Simon 1878).

Genus Buthus Leach 1815 (Leach 1815).

*Buthus saidnouirai* sp. *nov*.

**Etymology:** named after Professor Said NOUIRA, a Tunisian Herpetologist and Entomologist whose educational and scientific qualities, influenced generations of Tunisian academics and researchers in animal biology, significantly contributing to the improvement of knowledge about Tunisian fauna. His long‐standing and invaluable support to the first author (S.H.) and his contribution to the collection of studied scorpions were decisive for conducting the current work and for the description of this new species.

**Diagnosis:** Relatively large size, exceeding 70 mm in length. The body is yellowish‐brown, with prominent black carinae. The aculeus has almost the same length as the vesicle and its tip touches the vertical tangent passing through the vesicle's highest point. The carapace is slightly wider than long, marked by well‐protruding carinae. The granules on the ventral surface of the fifth segment's median carina increase in size from top to bottom.

**Description:** The measurements of the different body parts are given in Table 5. Coloration: **Prosoma** is yellowish‐brown with some dark brown to black traces (under carinae, around and between median eyes). Carinae is brown to slightly reddish (brown for the holotype). Eyes with dark green pigmentation. **Mesosoma** is yellowish‐brown with blackish bands: an axial band and well‐defined sagittal bands. Carinae is brown to slightly reddish (brown for the holotype). **Sternites** uniformly light yellowish‐brown. **Pectines and genital operculum** pale yellow **Metasoma** light yellowish‐brown to yellowish‐brown (light yellowish‐brown for the holotype) with some dark brown to black (dark brown for the holotype) in the dorsal median of segment I and slightly under the dorsal lateral carinae of segment I, ventral sub‐medians carinae of II–IV, and ventral median carinae of V. Carinae is brown to slightly reddish (brown for the holotype). **Telson** vesicle is light yellowish‐brown to yellowish‐brown (light yellowish‐brown for the holotype), aculeus is yellowish‐brown at its base, then a small part of reddish and then dark brown until its extremity. **Chelicerae** is light yellowish‐brown to yellowish‐brown (light yellowish‐brown for the holotype); fingers have dark brown teeth. **Pedipalps** is light yellowish‐brown to yellowish‐brown (light yellowish‐brown for the holotype); fingers' granules rows are dark brown. Legs are light yellowish‐brown in color (slightly lighter than pedipalps).

**TABLE 5:** Measurements of the prosoma, chela, telson, segments of the metasoma, numbers of pectinal teeth, and number of rows of granules of movable finger of *Saidnouirai* sp. *nov*.

	Holotype (female) (mm)	Allotype (male) (mm)
Prosoma length	77	56
Prosoma width	90	65
Chela length	131	99
Chela width	36	25
Chela height	33	22
Length of first metasoma segment	53	44
Width of first metasoma segment	53	44
Height first metasoma segment	47	36
Length of second metasoma segment	60	52
Width of second metasoma segment	51	43
Height second metasoma segment	48	37
Length of third metasoma segment	62	55
Width of third metasoma segment	50	41
Height third metasoma segment	47	36
Length of fourth metasoma segment	74	64
Width of fourth metasoma segment	48	39
Height fourth metasoma segment	43	32
Length of fifth metasoma segment	90	70
Width of fifth metasoma segment	47	37
Height fifth metasoma segment	37	27
Telson length	72	62
Telson width	37	29
Telson height	32	27
Pectinal teeth number (left–right)	26–26	30–31
Granules rows number	12	12

**FIGURE 10:**
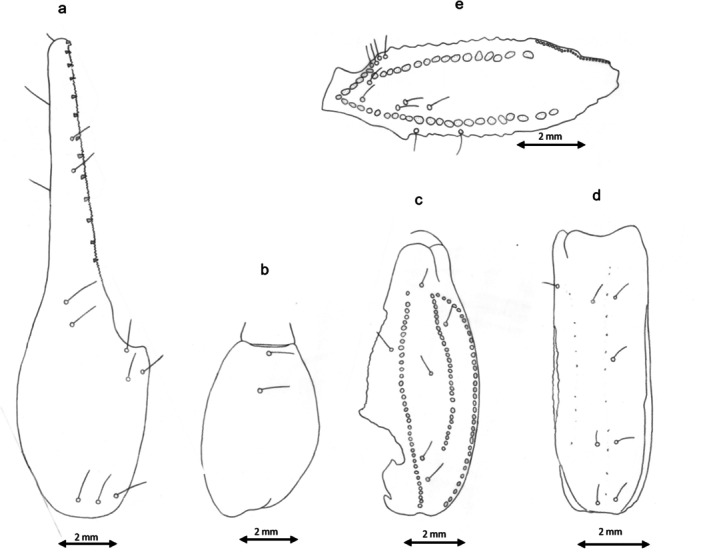
Trichobothrial pattern of *B. saidnouirai* sp. *nov*: (a) chela dorso‐external pattern; (b) chela ventral pattern; (c) patella dorsal aspect; (d) patella external pattern; (e) femur dorsal pattern.

**Morphology: The carapace** is trapezoidal in shape, slightly wider than long anterior margin straight to shallowly concave (shallowly concave for the holotype). Moderately granular. Well‐marked Carinae, typical of the genus, with moderately to strongly granulation and ‘lyre’ configuration. Moderate furrows. Median eyes are located in the center of carapace. They are more oval than circular and are separated by more than two ocular diameters. Lateral eyes are much smaller. **Sternum** is triangular and has very small m**esosoma** Three longitudinal carinae is moderately granulate in all tergites. Lateral part of tergites are moderate to strongly granulate (moderate granulation for the holotype) with big granules having all the same size. **Genital operculum** is divided longitudinally giving two semi‐oval plates. **Pectines: the** pectinal tooth count is 25–28 in females (26 for the holotype) and 30–34 in males. **Sternites have** four carinae on sternite VII; marked with small to mid‐sized granules, having all the same interior limit. The length of the interior carinae is slightly more than three‐quarters of the sternite length. **Metasoma** size is standard of the genus, progressively narrower and lower distally, with segment I having almost the same length and width. Segment I bearing 10 carinae, II–IV bearing eight, V bearing five. *dorsal laterals* moderately protruding and finely crenulate by small‐sized granules on I–IV, absent on V; *lateral supramedians* moderately protruding and finely crenulate by small‐sized granules on I–V; *lateral inframedians* moderately protruding and coarsely subcrenulate to granulate (subcrenulate for the holotype), complete on I, posterior two‐thirds of II and little more or less than posterior half of III, absent on IV and V; *ventral laterals* moderately protruding and coarsely subcrenulate to granulate (subcrenulate for the holotype) on I–V (V crenulate posteriorly with 3–4 lobes (four lobes for the holotype)); *ventral sub‐medians* moderately protruding and coarsely on I, well protruding and coarsely crenulate on II–III, protruding and coarsely crenulate with some spacing on IV, an incomplete row just some raised granules non‐ordered on V; *ventral median* is absent on I–IV, moderately protruding and almost regularly serrate increasing in size from top to bottom moderately on V (bifurcate on distal tip). Intercarinal tegument is weakly granular. **Telson:** vesicle is moderately bulbous and highly curved aculeus. The aculeus has almost the same length as the vesicle. The aculeus tip touches the vertical tangent passing through the vesicle's highest point. **Chelicerae**, the external and internal distal teeth have almost the same length. The movable fingers' basal teeth robust and not joined. **Pedipalps** consists of femur with 5 carinae; patella with 7–8 carinae (8 carinae for the holotype), moderately to lowery marked; Chela is smooth, large and robust. Movable and fixed fingers have 12 rows of granules; each one containing one internal and one external granule, and three distal granules. **Legs** are slender, weakly carinate, tibial and pedal spurs are big and thin. **Trichobothria** as defined by Vachon (1973). Trichobothrial pattern (Figure 10) type A, characteristic of the Buthidae family, orthobothriotaxic (Vachon 1973).

**Material examined:** Fifteen specimens were collected from various northern humid and coastal regions (Zouaraa, Bechouk, Ras Enjla, Haouaria, Zembra and Nfidha) of Tunisia between February 6, 2021, and June 4, 2022. Four females (including the holotype and three paratypes) and two males (including the allotype and a paratype) were deposited in the Scientific Collection of the Faculty of Sciences of Tunis under the references Sc17, Sc39, Sc21, Sc15, Sc37, and Sc52, respectively.

**Type locality:** The holotype was collected from Zembra (37°07′33.01″ N; 10°48′10.17″ E). The allotype was collected from Bechouk (37°06′59.80″ N; 9°32′13.16″ E). The paratypes were collected respectively from Bechouk (37°06′59.80″ N; 9°32′13.16″ E), Zembra (37°07′33.01″ N; 10°48′10.17″ E), Zembra (37°07′33.01″ N; 10°48′10.17″ E), and Hawaria (37°03′48.74″ N; 11°00′58.69″ E).

## 4 Discussion

Numerous recent studies have revealed the existence of an undescribed scorpion diversity that affects various groups, particularly in the Maghreb region including Morocco, Algeria and Tunisia (Khammassi et al. 2024). During the last 23 years, the number of new *Buthus* species described has increased significantly. The taxonomy of the genus *Buthus* becomes somewhat difficult due to the lack of neotypes for many species, the unclear distribution areas, and the discrepancies between descriptions in the identification keys for the same species (Vachon 1952; Levy and Amitai 1980; Lourenço 2002; Sousa et al. 2017). As a result, many taxonomists have advocated for additional research to stabilize the taxonomy within this genus (Sousa et al. 2017).

While there have been several recent molecular studies conducted in the Mediterranean region, the Iberian Peninsula and Morocco have received the most attention (Sousa et al. 2010; Pedroso et al. 2013; Klesser et al. 2020; Habel et al. 2012). The Tunisian region has been included in studies targeting the whole North Africa, with limited sample sizes employed more in a phylogenetic than a taxonomic context. Through the combination of barcoding and morphological assessment, our research allowed us to reexamine the taxonomy of the *Buthus* genus in Tunisia.

Our molecular findings match well with those previously established by Sousa et al. (2012) and Pedroso et al. (2013), while providing considerable new information. The deep branch (grouping C, D, E, F, and G) grouping all specimens from Tunisia with those from eastern Algeria corresponds to the same phylogenetic group (Clade D) defined by Sousa et al. (2012) as the “tunetanus” phylogenetic group. Further, using only 15 specimens from Tunisia, Pedroso et al. (2013) reported the existence of four subclades E2, E1, E4 and E3 occurring respectively in the extreme north, extreme south, and the top and the bottom of the Dorsal Mountains (the eastern part of the Atlas Mountain chain). With more specimens in our investigation, these four subclades were maintained, albeit with a more intricate geographic distribution of groups. With this new dataset, the distribution becomes respectively: the north and center of Tunisia (G4), the extreme south (G2), the Dorsal Mountain region (G3), and the forests of northern Tunisia including part of the Dorsal Mountains (G1). The divergence level between all these subclades exceeds the minimum species divergence threshold. Considering the limited vagility of *Buthus* species, the rare overlap of their distribution over the world (Gantenbein and Keightley 2004; Vahidinia et al. 2025) and previous descriptions of the Tunisian *Buthus* species distribution (or type localities), we consider that G1 corresponds to *B. paris* (occurring in the forests of northern Tunisia with the Dorsal Mountains), G3 corresponds to *B. chambiensis* (occurring in the east of the Tunisian Dorsal Mountain region near Chambi region), G4 corresponds to *B. tunetanus* (occurring in the north and the center of Tunisia). The distribution of G2 covers all the south, a region including the type locality of *B. dunlopi* . The large branch grouping all of these 4 subclades forms a lineage distinct from a new group reported in this work for the first time. This group exhibits high genetic differences from all other Tunisian subclades, more than double those between the different *Buthus* species already reported in Tunisia. It differs from the nearest lineage by 8%, a value typical of interspecific variation in the genus *Buthus*, and well above the highest reported intraspecific variation in well‐sampled species of *Buthus* from the Iberian Peninsula (Blasco‐Aróstegui et al. 2025). We propose that this new clade corresponds to a new species, *B. saidnouirai* sp. *nov*., inhabiting the country's northern and eastern coastal regions and wetlands including the island of Zembra.

While the ASAP approach suggests that the groups described above could potentially correspond to an even greater number of species, this method is known to “over‐split” low‐vagility, dispersal‐limited organisms like scorpions (e.g., Heine et al. 2024), and therefore, we maintain a more conservative approach of recognizing only these highly divergent lineages, which also show morphological cohesion. Under a modern principle of integrative taxonomy (Padial et al. 2010), these presumed species, identified by genetic criteria, need to be confirmed also by reliable morphological criteria. There are significant differences—sometimes even contradictions—between all the keys that have been developed since *Buthus* was originally described in Tunisia. For this reason, we performed a morphological characterization of the different clades obtained in order to review species discriminant criteria.

For the group corresponding to the distribution area of *B. paris*, the stocky shape of the carapace and their robust chela identified in this work was also mentioned by Koch (1839), as well as the high, protruding granulation, the segment metasomal size and the telson shape, which were mentioned previously by Vachon (1952). In addition to describing each taxonomically relevant body part, this work also presented characters exclusive to *B. paris*, differentiating it from the other four species. These characters are particularly the carapace line, the placement of the lateral eyes and the size of the first metasomal segment, which is wider than longer. For the group matching the *B. tunetanus* distribution area, the carapace granulation reported in this work as well as the chela shape and telson shape have also been described respectively by Koch (Simon 1878), Ehrenberg (Hemprich and Ehrenberg 1829) and Koch (Simon 1878), and Vachon (Vachon 1952), Lourenço (Lourenço 2003), and Kovarik (Kovařík 2006). Furthermore, the discriminating characters exclusive to *B. tunetanus* identified in this work are the mesosomal sagittal band, the anterior limit of the seventh sternal carinae and their length. As for *B. chambiensis*, the color, granulation, and sexual dimorphism described by Kovarik (Kovařík 2006) are also verified by our study. Additional discriminating criteria we propose for *B. chambiensis* are (1) exterior carinae that are higher than the interior ones at the seventh sternite, and (2) the specific granulation of the ventral median carinae of the fifth metasomal segment.

As Pedroso et al. (2013) and others have pointed out, the morphology of the groups that contain the type locality of *B. dunlopi* does not match perfectly with those reported in the original descriptions of Kovařík (2006). The original key was based only on the color of the body segments, size of some segments and the presence/absence of sexual dimorphism. However, for this last criterion, sexual dimorphism has since been demonstrated for all Tunisian *Buthus* (Hajri 2022; Hajri et al. 2024). Additionally, the criteria of pedipalp robustness in comparison to *B. tunetanus* and the number of granule rows are not verified. Moreover, our specimens are very different from the holotype and allotype shown in the pictures provided in the original description (Kovařík 2006). However, this group is characterized by movable fingers of pedipalp chela showing usually 11 rows of granules, elongated metasomal segments, first metasomal segment much longer than wide, carinae extremely less marked, and tergites uniformly light. These criteria better match the description of *B. lourencoi* Rossi, Tropea & Yağmur, 2013, a species reported from Tripoli in northern Libya (Rossi et al. 2013). We therefore suggest that the geographic range of *B. lourencoi* extends probably to southern Tunisia, but we believe that further studies are necessary to verify this hypothesis.

For the new species *B. saidnouirai* sp. nov., exclusive morphological characters are the increase in granule size from top to bottom of the ventral median carinae of the fifth metasomal segment, and the tip of the aculeus touching the tangent. In combination, the described criteria provided in this work readily distinguish each of the five species from each other.

Interestingly, the pattern of genetic divergence highlighted in our study is not related to morphological divergence, since the five described species show a clear morphological gradient concerning most of the criteria studied, notably the level of protruding granulation, the number of rows of mobile granules on the fingers, the shape of the telson, and the size of the first metasomal segment. *B. paris* and the *Buthus* inhabiting the south are the extremes of this gradient, while the new species, *B. saidnouirai* sp. *nov*, occupies an intermediate position. This morphological gradient reflects a north–south gradient of habitat conditions, which consists of high‐density forest habitats in the northwest, less dense forests eastwards (a region delimited to the northeast and east by the Mediterranean Sea), steppe habitats in the center, and desert habitats in the south (Hezzi 2014; Carlos 2018). Owing to this habitat gradient, scorpion populations are likely to inhabit areas with different prey, predator and competitor communities, which could expose their morphological traits, notably the size of chela, telson, and carapace, to different selective pressures, inducing the evolution of these traits in varied trajectories. Indeed, these body parts are known to be under high selective pressure, because they are used both in defense and hunting purposes (Coelho et al. 2018; Van Der Meijden et al. 2012; Simone and Van Der Meijden 2021). Regarding the gradient of ornamentation, it seems that *Buthus* species try to mimic the substrate's hue to make themselves less noticeable to predators. The ornamentation is darkest and most obvious in the densest forest substrates (*B. paris*), which are covered with leaves of different colors, cork, and stones. Then, it becomes lighter and lighter further south with the other species, as the substrate is covered with a few leaves and stones near the coast, little cover in the steppes and pure sand in the deserts (southern species).

In line with this, it has been suggested that the diverse topography and orographic variability of the Mediterranean region have played a primary role leading to speciation within the genus *Buthus* (Schoville et al. 2012). The topography and orography of the Tunisian territory are well matched with the geographic distribution of the reported phylogenetic entities. The Tunisian Chotts, a permanent saltwater expanse situated in a succession of tectonically controlled depressions of the pre‐Sahara region between the Atlas and Saharan platforms, can explain the delimitation of the ranges of the southern species corresponding probably to *B. lourencoi* and *B. tunetanus* (Deroin et al. 2009). These chotts have a known role as geographical barriers in numerous taxa, including other scorpions of the genus *Androctonus* (Ben Othmen et al. 2009). Furthermore, the northwestern mountainous forests of the country are home to *B. paris*. This species is mainly distributed in the Kroumirie, a group of mountainous massifs belonging to the northern Tell, an area known for its high rainfall, humid weather, and dense deciduous forest cover (Stambouli‐Essassi et al. 2007). *B. paris* occurs also in the western‐central region with *B. chambiensis*, a species endemic to this region, although without any records of sympatry (Kovařík 2006). It is a mountainous area of great geological importance, being the eastern extension of the Atlas Mountains separating the Tell from southern Tunisia (Arfaoui et al. 2016). It is also known as the island of Kasserine, which rose during the Cretaceous‐Eocene and served as a refuge for many of the region's land animals during the submergence of most of Tunisia by the Tethys Sea (Zouaghi et al. 2005). This geological history may be behind the great specific richness of this region and its high degree of endemism (Hewitt 2011). The new species is linked to humid environment, a newly prospected habitat type. It is distributed along the north and east coasts and near the rivers and sebkhas, including the island of Zembra. These are very humid habitats with mild winters, where daytime and night‐time temperatures are more similar due to the sea's moderating effect (Velea et al. 2012).

Interestingly, a single specimen from the western Mediterranean coastal region of Egypt appears here as an individual belonging to *B. saidnouirai* sp. nov. This Egyptian region is a well‐defined biogeographic area, and following (Badry et al. 2017), includes two known species of *Buthus*, *Buthus orientalis* Lourenço and Simon (2012) and *Buthus adrianae* Rossi (2013). These two species are distinguished by the presence of 10 rows of granules on the movable and fixed fingers, by an elongated metasoma, with all metasoma segments longer than wide. In addition, *Buthus adrianae* and *B. orientalis* are characterized respectively by a darkened carapace posterior part and a slender chela for both sexes, and are therefore clearly distinct from *B. saidnouirai* sp. *nov*. It appears therefore that *B. saidnouirai* sp. *nov*. extends beyond the Tunisian coasts and wetlands, and highlights the need for further taxonomic research on this genus throughout North Africa to precisely define its distribution range.

To conclude, our study reinforces the distinction of *B. tunetanus* , *B. paris*, and *B. chambiensis*, while we found no evidence of *B. dunlopi* in the studied samples. The possibility of occurrence of *B. lourencoi* in Tunisia is proposed here for the first time. Further, an additional lineage with distinct morphological and genetic characteristics that is considered a new fifth species, is described in this work, *B. saidnouirai* sp. *nov*. Additional lineages, known previously only from Algeria, may also occur on the fringes of Tunisia near the Algerian border. Our sampling better delimits the range of the five widespread Tunisian species, while our morphological assessment provides additional characters that can be used in their identification. Further taxonomic work is still needed to delimit the Algerian forms, while additional genetic markers would be needed to refine the placement of the Tunisian species within the phylogeny of the *Buthus* genus.

## Author Contributions

**Sarra Hajri:** data curation (equal), formal analysis (equal), investigation (equal), methodology (equal), software (equal), writing – original draft (equal). **Lilia Bahri:** conceptualization (equal), supervision (equal), writing – review and editing (equal). **David James Harris:** conceptualization (equal), formal analysis (equal), supervision (equal), writing – review and editing (equal).

## Funding

The authors have nothing to report.

## Conflicts of Interest

The authors declare no conflicts of interest.

## Supporting information


**Appendix S1:** ece372556‐sup‐0001‐AppendixS1.docx.

## Data Availability

All data generated or analyzed during this study are included in this published article and its Supporting Information files.
