# Supplementary material for: New Insights on Genetic and Morphological Divergence Among a Buthus Species Complex From Tunisia With the Identification of a New Species
Source: Ecol Evol. 2025 Nov 30;15(12):e72556. doi: 10.1002/ece3.72556 (PMC12665361; doi:10.1002/ece3.72556)
Supplement: Supplementary file 1 — Appendix S1: ece372556‐sup‐0001‐AppendixS1.docx. [file ECE3-15-e72556-s001.docx]

**Supplementary material:**

Table S1: Overview of specimen collected (providing sequences name, accession numbers (GenBank Id), exact geographic location, country and name of location, new specimen or from other reference, specimen is studied morphologically or only with COI and its position in the phylogenetic tree).

| Specimen  ID | GenBank  code | Station | Georeferenced details | | New / from other reference | Type of study | Clade |
| --- | --- | --- | --- | --- | --- | --- | --- |
|  |  |  | Latitude | Longitude |  |  |  |
| Sc2 | PX529891 | Tunisia (Khroufa) | 36.9333667 | 8.942275 | New | COI + Morphology | G1 |
| Sc3 | PX529892 | Tunisia (Khroufa) | 36.9333667 | 8.942275 | New | COI + Morphology | G1 |
| Sc4 | PX529893 | Tunisia (Khroufa) | 36.9333667 | 8.942275 | New | COI + Morphology | G1 |
| Sc5 | PX529894 | Tunisia (Khroufa) | 36.9333667 | 8.942275 | New | COI + Morphology | G1 |
| Sc6 | PX529895 | Tunisia (Khroufa) | 36.9333667 | 8.942275 | New | COI + Morphology | G1 |
| Sc7 | PX529896 | Tunisia (Khroufa) | 36.9333667 | 8.942275 | New | COI + Morphology | G1 |
| Sc8 | PX529897 | Tunisia (Khroufa) | 36.9333667 | 8.942275 | New | COI + Morphology | G1 |
| Sc9 | PX529898 | Tunisia (Khroufa) | 36.9333667 | 8.942275 | New | COI + Morphology | G1 |
| Sc10 | PX529899 | Tunisia (Khroufa) | 36.9333667 | 8.942275 | New | COI + Morphology | G1 |
| Sc11 | PX529900 | Tunisia (Khroufa) | 36.9333667 | 8.942275 | New | COI + Morphology | G1 |
| Sc12 | PX529901 | Tunisia (Khroufa) | 36.9333667 | 8.942275 | New | COI + Morphology | G1 |
| Sc13 | PX529902 | Tunisia (Zouaraa) | 37.0344167 | 8.929211 | New | COI + Morphology | D |
| Sc14 | PX529903 | Tunisia (Zembra) | 37.1253806 | 10.80282 | New | COI + Morphology | D |
| Sc15 | PX529904 | Tunisia (Zembra) | 37.1253806 | 10.80282 | New | COI + Morphology | D |
| Sc16 | PX529905 | Tunisia (Zembra) | 37.1253806 | 10.80282 | New | COI + Morphology | D |
| Sc18 | PX529906 | Tunisia (Zembra) | 37.1253806 | 10.80282 | New | COI + Morphology | D |
| Sc19 | PX529907 | Tunisia (Zembra) | 37.1253806 | 10.802825 | New | COI + Morphology | D |
| Sc20 | PX529908 | Tunisia (Zembra) | 37.1253806 | 10.802825 | New | COI + Morphology | D |
| Sc22 | PX529909 | Tunisia (Jedelienne) | 35.58015 | 9.0550166 | New | COI + Morphology | G3 |
| Sc23 | PX529910 | Tunisia (Jedelienne) | 35.58015 | 9.0550166 | New | COI + Morphology | G3 |
| Sc24 | PX529911 | Tunisia (Jedelienne) | 35.58015 | 9.0550166 | New | COI + Morphology | G1 |
| Sc25 | PX529912 | Tunisia (Jedelienne) | 35.58015 | 9.0550166 | New | COI + Morphology | G3 |
| Sc26 | PX529913 | Tunisia (Sbiba) | 35.5424417 | 9.0820305 | New | COI + Morphology | G4 |
| Sc27 | PX529914 | Tunisia (Kesra) | 35.8116806 | 9.3650472 | New | COI + Morphology | G3 |
| Sc28 | PX529915 | Tunisia (Pined Kef) | 36.1433056 | 8.7368833 | New | COI + Morphology | G1 |
| Sc29 | PX529916 | Tunisia (Dir Kef) | 36.1433056 | 8.7368833 | New | COI + Morphology | G1 |
| Sc30 | PX529917 | Tunisia (Ras Enjla) | 37.3393194 | 9.7484416 | New | COI + Morphology | D |
| Sc31 | PX529918 | Tunisia (Sidi Bouzid) | 35.0266556 | 9.4621444 | New | COI + Morphology | G4 |

| Sc34 | PX529919 | Tunisia (Mateur) | 37.0297972 | 9.67738611 | New | COI + Morphology | D |
| --- | --- | --- | --- | --- | --- | --- | --- |
| Sc35 | PX529920 | Tunisia (Mateur) | 37.0297972 | 9.67738611 | New | COI + Morphology | G1 |
| Sc39 | PX529921 | Tunisia (Bechouk) | 37.1166111 | 9.53698888 | New | COI + Morphology | D |
| Sc40 | PX529922 | Tunisia (Bechouk) | 37.1166111 | 9.53698888 | New | COI + Morphology | D |
| Sc41 | PX529923 | Tunisia (Sidi Bou Zid) | 35.0266556 | 9.4621444 | New | COI + Morphology | G4 |
| Sc43 | PX529924 | Tunisia (Tbaga) | 33.7061083 | 9.3928861 | New | COI + Morphology | G2 |
| Sc45 | PX529925 | Tunisia (Sidi Toui) | 32.7289333 | 11.230855 | New | COI + Morphology | G2 |
| Sc51 | PX529926 | Tunisia (Haouaria) | 37.0635389 | 37.0635389 | New | COI + Morphology | D |
| Sc52 | PX529927 | Tunisia (Haouaria) | 37.0635389 | 37.0635389 | New | COI + Morphology | D |
| Sc54 | PX529928 | Tunisia (Nfidha) | 36.1845667 | 10.435416 | New | COI + Morphology | D |
| Sc55 | PX529929 | Tunisia (Sidi Toui) | 32.7289333 | 11.230855 | New | COI + Morphology | G2 |
| Sc58 | PX529930 | Tunisia (Kasserine) | 35.1845917 | 8.833575 | New | COI + Morphology | G3 |
| Sc59 | PX560079 | Tunisia (Zouaraa) | 37.0344167 | 8.929211 | New | COI + Morphology | D |
| Sc956 | PX529931 | Egypt | 31.279399 | 27.054969 | New | COI + Morphology | D |
| Ho111 | MT955945 | Algeria | 22.942631 | 5.592652 | Klesser et al. (2021) | COI | A |
| Ho181 | MT955943 | Algeria | 23.017225 | 5.341339 | Klesser et al. (2021) | COI | A |
| Ho182 | MT955944 | Algeria | 23.017225 | 5.341339 | Klesser et al. (2021) | COI | A |
| JN52 | JN885952 | Algeria | 29.49 | -5.38 | Klesser et al. (2021) | COI | B |
| JN53 | JN885953 | Algeria | 29.49 | -5.38 | Klesser et al. (2021) | COI | B |
| Sc370 | JQ775953 | Algeria | 36,832 | 4,090 | Sousa et al. (2012) | COI | E |
| Sc373 | JQ775954 | Algeria | 36,472 | 4,007 | Sousa et al. (2012) | COI | E |
| Sc375 | JQ775959 | Algeria | 35.302 | 7.652 | Sousa et al. (2012) | COI | C |
| Sc402 | JQ775958 | Algeria | 35,582 | 6,063 | Sousa et al. (2012) | COI | F |
| Sc403 | KF824991 | Algeria | 35.581 | 6.063 | Sousa et al. (2012) | COI | E |
| Sc405 | JQ775959 | Algeria | 35,398 | 1,332 | Sousa et al. (2012) | COI | D |
| Sc407 | JQ775960 | Tunisia (Djerba) | 33,847 | 10,831 | Sousa et al. (2012) | COI + Morphology | G4 |
| Sc408 | JQ775961 | Tunisia (Djerba) | 33,847 | 10,831 | Sousa et al. (2012) | COI + Morphology | G4 |
| Sc409 | JQ775962 | Tunisia (Zelfene) | 35,483 | 8,744 | Sousa et al. (2012) | COI | G4 |
| Sc410 | JQ775963 | Tunisia (Zelfene) | 35,483 | 8,744 | Sousa et al. (2012) | COI + Morphology | G4 |
| Sc411 | JQ775964 | Tunisia (Kbouch) | 36,212 | 8,900 | Sousa et al. (2012) | COI + Morphology | G1 |
| Sc412 | JQ775965 | Tunisia (Kbouch) | 36,212 | 8,900 | Sousa et al. (2012) | COI + Morphology | G1 |
| Sc413 | JQ775966 | Tunisia (Kbouch) | 36,212 | 8,900 | Sousa et al. (2012) | COI | G1 |

| Sc891 | KF825010 | Tunisia (Ghesala) | 37.070 | 9.496 | Pedroso et al. (2013) | COI + Morphology | G1 |
| --- | --- | --- | --- | --- | --- | --- | --- |
| Sc893 | KF825011 | Tunisia (Fajj al Tamir) | 35.882 | 8.712 | Pedroso et al. (2013) | COI + Morphology | G1 |
| Sc894 | KF825012 | Tunisia (Fajj al Tamir) | 35.882 | 8.712 | Pedroso et al. (2013) | COI + Morphology | G1 |
| Sc897 | KF825013 | Tunisia (Thala) | 35.554 | 8.681 | Pedroso et al. (2013) | COI + Morphology | G4 |
| Sc898 | KF825014 | Tunisia (Thala) | 35.554 | 8.681 | Pedroso et al. (2013) | COI + Morphology | G4 |
| Sc900 | KF825015 | Tunisia (Majal Bel Abbas) | 34.711 | 8.516 | Pedroso et al. (2013) | COI | G4 |
| Sc901 | KF825016 | Tunisia (Majal Bel Abbas) | 34.711 | 8.516 | Pedroso et al. (2013) | COI + Morphology | G4 |
| Sc906 | KF825017 | Tunisia (Gafsa Sud) | 34.333 | 8.578 | Pedroso et al. (2013) | COI + Morphology | G4 |
| Sc907 | KF825018 | Tunisia (Gafsa Sud) | 34.333 | 8.578 | Pedroso et al. (2013) | COI + Morphology | G4 |
| Sc930 | KF825019 | Tunisia (Matmata) | 33.532 | 9.990 | Pedroso et al. (2013) | COI + Morphology | G2 |
| Sc941 | KF825020 | Tunisia (Dar Souid) | 32.785 | 10.373 | Pedroso et al. (2013) | COI + Morphology | G2 |
| Sc942 | KF825021 | Tunisia (Dar Souid) | 32.785 | 10.373 | Pedroso et al. (2013) | COI + Morphology | G2 |
| Sc943 | KF825022 | Tunisia (Dar Souid) | 32.785 | 10.373 | Pedroso et al. (2013) | COI + Morphology | G2 |
| Sc945 | KF825023 | Tunisia (Tataouine North) | 32.899 | 10.250 | Pedroso et al. (2013) | COI + Morphology | G2 |
| TU1 | AJ506915 | Tunisia (Sidi Amor Bou  Hajla) | 35,361 | 10,115 | Gantenbein (2003) | COI | G4 |
| TA1 | AJ506916 | Tunisia (Dgueche) | 32,523 | 8,054 | Gantenbein (2003) | COI | C |
| TA2 | AJ506917 | Tunisia (Om Al-Araies) | 32,619 | 8,135 | Gantenbein (2003) | COI | G4 |
| TA3 | AJ506918 | Tunisia (Assabika) | 32,618 | 8,054 | Gantenbein (2003) | COI | G4 |
| F1 | MT955935 | Tunisia (Ain Draham) | 36.45 | 8.44 | Klesser et al. (2021) | COI | G4 |
| F3 | MT955936 | Tunisia (Ain Draham) | 36.45 | 8.44 | Klesser et al. (2021) | COI | G4 |
| F5 | MT955934 | Tunisia (Ain Draham) | 36.45 | 8.44 | Klesser et al. (2021) | COI | G4 |
| Sc0287 | KF825025 | Morocco | 32.661 | -7.792 | Sousa et al. (2012) | COI | Outgroup |

6.5 3.0 4.0 2.0 5.0 7.5 6.0 5.5 12.0 10.0

**ASAP SCORE**


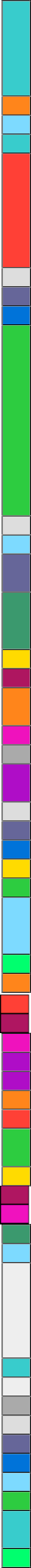

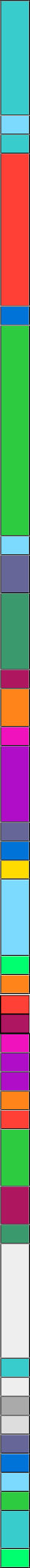

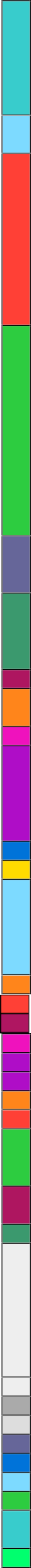

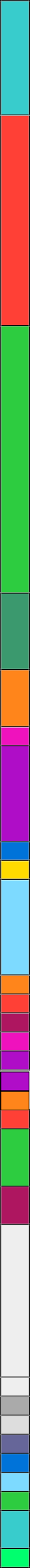

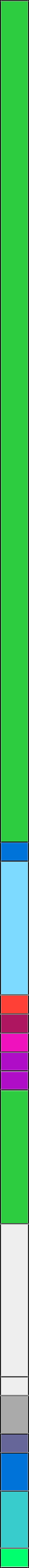

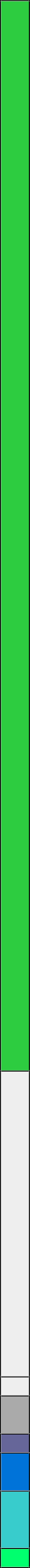

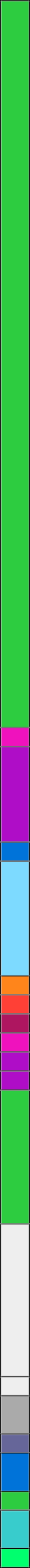

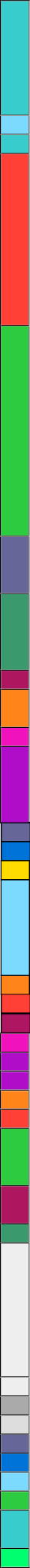

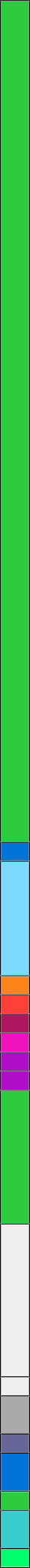

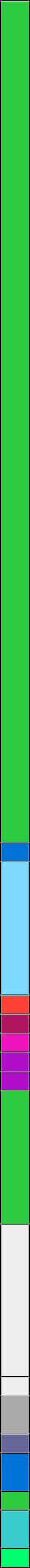
F5 F3

Sc410 Sc0898 Sc0897 F1 TA3 TA2 Sc41 Sc409 Sc26 Sc0906 Sc0901 Sc0900 Sc31 Sc0907 Sc407

Sc9 Sc8 Sc6 Sc5 Sc4 Sc3 Sc2 Sc12 Sc11 Sc10

Sc7 Sc35 Sc24 Sc0891 Sc413 Sc412 Sc28 Sc411 Sc29 Sc0894 Sc0893 Sc408 Sc58 Sc23 Sc22 Sc25 Sc27 Sc55 Sc45 Sc0945 Sc0943 Sc0942 Sc0941 Sc0930 Sc43 Sc403 Sc402 TA1 SC375 Sc956 Sc59 Sc13 Sc54 Sc34 Sc30 Sc40 Sc39 Sc52 Sc51 Sc19 Sc16 Sc15 Sc14 Sc18 Sc20 Sc405 Sc373 Sc370 TU1 JN53 JN52 Ho111 Ho182 Ho181 Sc0287

Figure S1: ASAP Output
